# Effect of Superheat Steam on Ejector in Distilled Water Preparation System for Medical Injection

**DOI:** 10.3390/e24070960

**Published:** 2022-07-11

**Authors:** Bin Yang, Xiaojing Ma, Hailun Zhang, Wenxu Sun, Lei Jia, Haoyuan Xue

**Affiliations:** 1 Institute of Marine Science and Technology, Shandong University, Qingdao 266237, China; 202036957@mail.sdu.edu.cn (B.Y.); maxiaojing@sdu.edu.cn (X.M.); 202199900086@sdu.edu.cn (H.Z.); 202290900028@sdu.edu.cn (W.S.); 2 School of Control Science and Engineering, Shandong University, Jinan 250061, China

**Keywords:** ejector, non-equilibrium condensation, superheat steam, computational fluid dynamics

## Abstract

In this study, a wet steam model was used to investigate the effect of steam superheat on ejector performance and non-equilibrium condensation phenomena. The simulation data for the ejector were validated with experimental data. The simulations show that an increase in primary flow superheat will increase the entrainment ratio, while an increase in secondary flow superheat will decrease the entrainment ratio. The output fluid superheat has little effect on the entrainment ratio. As the primary flow superheat increases from 0 to 20 K, the starting position of non-equilibrium condensation moves backward by 5 mm, and the mass fraction of condensed droplets decreases by 20%. The higher the secondary flow superheat, the lower the mass fraction of liquid in the diffusion chamber. The superheat level of the output fluid has no influence on the non-equilibrium condensation phenomenon of the ejector.

## 1. Introduction

A supersonic ejector is an energy conversion device that uses a high-pressure dynamic gas to eject low-pressure gas. It has been widely employed in seawater desalination, refrigeration, pharmaceutical systems, and other areas [1,2], owing to its simple structure, low cost, and excellent stability. The supersonic ejector is the key component for accomplishing energy cascade utilization and lowering energy consumption in distilled water preparation systems. The water production efficiency of the distilled water preparation system for medical injection is associated with the performance of the ejector and operating stability [3]. Accurate ejector performance analysis and prediction can significantly improve the system performance and stability, increase the steam cycle rate of a distilled water preparation system, and significantly reduce enterprise water production costs.

The construction and performance of ejectors have been thoroughly studied. According to the position of the nozzle exit, Keenan et al. [4] classified the ejector as being either “constant-area mixing” or “constant-pressure mixing.” Because it is widely assumed that the constant-pressure mixing ejector has greater stability and a wider range of backpressures, it has been the subject of many subsequent studies. Huang [5] assumed that the two fluids were mixed in the constant-pressure mixing chamber and developed a one-dimensional model for the double-choking constant area mixing process. Chen [6] proposed a one-dimensional model for predicting ejector performance under critical and sub-critical conditions. Huang [7] tested and analyzed 15 ejectors and developed two empirical formulas for predicting ejector performance with R141b as the working fluid. El-Dessouky [8] developed a semi-empirical model for the design and evaluation of ejectors. Ruangtrakoon [9] presented a theoretical calculation method for designing the steam ejectors for refrigeration applications, the results showed that this calculation method is highly effective. Tashtoush [10] proposed two 1-D ejector mathematical models to predict the performance of a solar ejector cooling system, the study showed that the solar ejector cooling system with a variable area ejector has a higher coefficient of performance under a vast range of operational conditions. Megdouli [11] found that the new combined refrigeration cycle had a lower optimum gas cooler pressure, higher coefficient of performance, and higher exergy efficiency. Zeng [12] improved the coefficient of performance about 42% on the dual-ejector transcortical CO_2_ cycle for low-temperature refrigeration by means of multi-parameter optimization. Friso [13] provided an algorithm to determine the maximum value of the entrainment ratio.

The thermodynamic and empirical formula models mentioned in the literature require many assumptions and are incapable of describing the fluid state and complex physical mixing processes in the ejector. Computational fluid dynamics (CFD) can simulate complex processes such as turbulence, shock waves, and non-equilibrium condensation in an ejector by two-dimensional or three-dimensional meshes. Early CFD simulations were limited by computer capability, and the coarse meshes used could not accurately predict the internal flow field and performance of the ejector [14]. With the rapid development of computer science, CFD simulations have gradually been favored for ejector modeling and performance optimization. CFD simulations cannot only accurately predict the performance of the ejector [15] but also reflect complex mixing processes such as choking flow, oblique shock waves, and non-equilibrium condensation in the ejector [16]. Rusly [17] analyzed the dynamic flow using CFD simulations to obtain more accurate predicted data. Tashtoush et al. [1] described the ejector design approach and discussed the influence of geometrical parameters. Wang [18] presented a comprehensive numerical model of the ejector’s supersonic flow which shows that the primary pseudo-shock flow pattern plays a critical role in determining the ejector performance. Many studies have revealed that the two most critical structural elements influencing ejector performance are the nozzle exit position (NXP) and the area ratio (AR) of the constant area mixing chamber to the nozzle throat. Pianthong et al. [19] discovered that moving the nozzle exit position closer to the primary inlet improves the effective area of the hypothesized throat and ejector wall, improving the entrainment ratio; however, moving it too close causes a loss of primary flow and reduces the entrainment ratio. Yan [20] also discovered that AR and NXP were the two key structural factors influencing the entrainment ratio using CFD modeling. Carrillo [21] optimized the performance of a single-phase ejector by means of a Multi-Objective Evolutionary Algorithm coupled with a surrogate model based on CFD simulations.

As research has developed, the condensation phase modification of high-speed fluids in ejectors has become a key focus of academic interest. Cai [22] discovered that the maximum Mach number of non-equilibrium condensation was greater than that of equilibrium condensation under no-slip conditions between the phases. Ariafar [23] employed the wet steam model to obtain a higher entrainment ratio and critical backpressure than those of the ideal gas model. Wang [24] discovered that increasing the superheat in the primary nozzle causes the condensation shock wave to move backward, the liquid mass fraction to decrease, and the entropy generation to increase. Yang [25] compared the wet steam model with the dry gas model and found that the dry gas increased the expansion characteristics. In the case of insufficient primary flow expansion, the entrainment ratio of the dry gas model was greater than that of the wet steam model. In the case of sufficient expansion of primary flow, the entrainment ratio of the dry gas model was smaller than that of the wet steam model. Tang [26] investigated the condensation and re-evaporation processes of steam ejectors using high-speed camera-imaging technology.

The superheat of steam at the ejector port changes the internal non-equilibrium condensation phenomenon, impacting the performance of the ejector and operating conditions. Wang [27] discovered that increasing the superheat of the primary flow in critical mode may enhance the entrainment ratio in an ejector refrigeration system. Wang [28] discovered that using superheated wet steam as the primary flow reduces the condensation intensity and delays the condensation location. Zhang [29,30] investigated the non-equilibrium condensation in an ejector and discovered that wet steam was more consistent with real gas than the ideal gas model. The use of superheated steam would reduce energy consumption and enhance the entrainment ratio, whereas lowering the secondary flow temperature would also improve the entrainment ratio.

The experimental data show that the superheat phenomenon of steam sometimes occurs in the three ports of the ejector throughout the operation process in the distilled water preparation system for medical injection. The superheating phenomenon affects the condensation process inside the ejector and changes the ejector performance. The ejector performance is related to the normal operation of the entire system. However, there are few relevant research reports on this phenomenon. Many researchers focused on the structural design and optimization, such as the primary nozzle of ejector, the secondary flow port, and mixed flow outlet. Hence, in order to take advantage of the superheating in the system and suppress its disadvantages, this study evaluates the effects of superheat steam on three ports of an ejector in a distilled water preparation system based on the operating status and data.

## 2. Ejector

**Figure 1:**
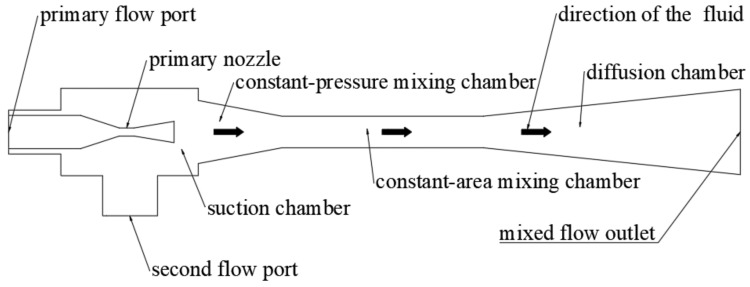
Ejector structure.

The supersonic ejector is composed of five parts: the primary nozzle, suction chamber, constant-pressure mixing chamber, constant-area mixing chamber, and diffusion chamber. Its structure is shown in Figure 1. The high-pressure primary fluid expands rapidly in the primary nozzle and converts the pressure potential energy into kinetic energy, which causes the primary flow to gradually transform from a subsonic to supersonic fluid and forms a low-pressure vacuum area at the exit of the primary nozzle. The low-pressure secondary flow is injected into the constant-pressure mixing chamber to accelerate the expansion and reach the choke state in the constant-area mixing chamber owing to the suction effect in the low-pressure vacuum zone and the shear banding effect of the high-speed fluid. Subsequently, the two fluids were mixed in the constant-area mixing chamber, and the primary and secondary flows exchanged energy and momentum. The velocity of the mixed fluid eventually tended to be consistent during the flow along the ejector, and the mixing of the primary and secondary flows was complete. The kinetic energy was then converted into pressure potential energy by the mixed flow, owing to the pressure-boosting effect in the diffusion chamber. 

**Figure 2:**
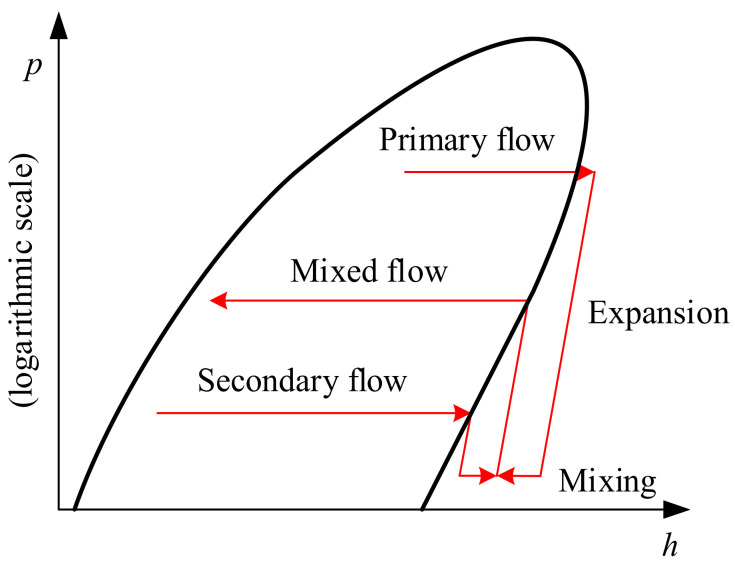
Pressure-enthalpy diagram of the distilled water preparation system.

The pressure-enthalpy diagram of distilled water preparation system is shown in Figure 2. As the high-pressure steam flows through the ejector, it converts some of its enthalpy into a low-pressure secondary steam. As a result of the energy transfer, the secondary fluid is compressed. The two streams mix and leave the ejector at the intermediate pressure. The non-equilibrium condensation phenomenon occurs in this process, which increases the cooling and depressurization to achieve supersaturation after the water vapor enters the Laval nozzle. Subsequently, the water vapor undergoes a phase transition, and a condensation nucleus is generated at the outlet of the Laval nozzle. The condensation nucleus increases progressively as the high-speed fluid passes through a supercooled environment, while the pressure and temperature rise and the fluid velocity declines. The condensed droplets re-evaporated into water vapor. The entrainment ratio (ER), which is defined as the ratio of the secondary inlet mass flow to the primary inlet mass flow, is the most essential performance indicator of the ejector. The droplet nucleation rate is the number of nuclei formed per unit time and volume. The liquid mass fraction in a fluid is the ratio of the liquid mass to the gas-liquid two-phase flow mass per unit volume.

The multi-effect distillation process is used in the distilled water preparation system for medical injection. Based on the operating environment of the ejector and the design theory of the ejector, the structural parameters of the ejector are calculated as shown in Table 1. Because the operating circumstances of the system vary within a range, when the primary steam pressure is insufficient, the streamlined primary nozzle can achieve a greater entrainment ratio than the normal primary nozzle [31]. As a result, the primary flow nozzle in this study was designed in a streamlined manner.

## 3. Numerical Procedure

### 3.1. Wet Steam Model

The physical properties of wet steam as well as the phase change properties have been captured very precisely, but many empirical formulations of wet steam state characteristics are too difficult to use in numerical simulation. As a result, the virial equation of the state of Young wet steam was used in this study, the equation of which is that pressure is simply related to temperature and density [32].

$$P=\rho RT(1+B\rho +C\rho^{2}+\ldots )$$

This method has two advantages. First, the virial coefficients *B* and *C* are only related to temperature. Second, this equation can be shortened at any point as long as the accuracy matches the requirements.

The virial equation of state for the general use of wet steam is:

(1) $$P=\rho RT(1+B\rho +C\rho^{2})$$

where ρ denotes the density, *R* denotes the specific gas constant, T denotes the thermodynamic temperature. The second virial coefficient B and the third virial coefficient *C* are empirical expressions.

(2) $$B=a_{1}{\left(1+\frac{T}{a}\right)}^{-1}+a_{2}e^{t}{\left(1-e^{-t}\right)}^{\frac{5}{2}}t^{-\frac{1}{2}}+a_{3}t$$

The unit of *T* in the above empirical expression is K, the unit of *B* is m^3^/kg, and the other parameters have the following empirical conditions.

$$\begin{array}{l} \tau =1500/T;\\ a_{1}=0.0015;\;\alpha =10,000;\\ a_{3}=-0{.0004882;\;a}_{2}=-0.000942 \end{array}$$

The third-dimensional coefficient is the empirical expression:

(3) $$C=a\left({\varepsilon -\varepsilon }_{0}\right)e^{-\eta \varepsilon }+b$$

where ε=T/647.286. *T* is the temperature in unit of K; the unit of *C* is m^6^/kg^2^; other relevant empirical coefficients have the following relationships:

$$\begin{array}{l} \varepsilon_{0}=0.8978;\;a=1.772;\\ \eta =11.16;\;b=1.5\times 10^{-6} \end{array}$$

### 3.2. Non-Equilibrium Condensation Phase Transition Model

The phase transition process of wet steam includes droplet nucleation and droplet growth, which can be described by the following formula [33]:

(4) $$\begin{array}{c} \frac{\partial \left(\rho y\right)}{\partial t}+\frac{\partial \left({\rho yu}_{j}\right)}{\partial x_{j}}=\dot{m} \end{array}$$

(5) $$\frac{\partial \left(\rho n\right)}{\partial t}+\frac{\partial \left({\rho nu}_{j}\right)}{\partial x_{j}}=\rho J$$

where $\dot{m}$ and *J* represent the droplets growth rate and nucleation rates, respectively.

(6) $$\begin{array}{c} \dot{m}\;=\frac{{4\pi r}^{*}{}^{3}}{3}\rho_{l}{J+4\pi \rho }_{l}{nr}^{2}\frac{dr}{dt} \end{array}$$

(7) $$\begin{array}{c} n=\frac{\beta }{\left(1-\beta \right)V_{d}(\rho_{l}/\rho_{v})} \end{array}$$

where $\rho_{l}$ denotes the droplet density, *r* denotes the droplets radius, r* denotes the critical radius, $\frac{dr}{dt}$ denotes the droplet growth rate, *n* denotes the number of droplets per unit volume, and β denotes the liquid mass fraction.

The nonisothermal classical nucleation theory for the droplets’ nucleation rate is given by:

(8) $$\begin{array}{c} J=\frac{q_{c}}{1+\varphi }\frac{\rho_{v}{}^{2}}{\rho_{l}}\sqrt{\frac{2\sigma }{{\pi m}_{v}{}^{3}}}\exp\left(-\frac{4\pi \sigma }{{3k}_{B}T_{v}}r^{*}{}^{2}\right) \end{array}$$

where $q_{c}$ is the condensation efficiency, φ is the correction coefficient, $m_{v}$ is the mass of the gas molecule, and $k_{B}$ is the Boltzmann constant.

Calculation formula of droplets growth rate is:

(9) $$\begin{array}{c} \frac{dr}{dt}=\frac{\lambda_{v}\left(T_{S}{-T}_{V}\right)}{\rho_{l}{r(h}_{v}{-h}_{l})}\frac{1-\frac{{\;r}^{*}}{r}}{\left(1+3{.18K}_{n}\right)} \end{array}$$

where $K_{n}$ is the Knudsen number of the liquid [34].

### 3.3. Governing Equations

The internal pressure, temperature, velocity, and other variables were primarily characterized in computational fluid dynamics by mass conservation, energy conservation, and momentum conservation. Some ideal assumptions are needed in the study because the mixing process of the two streams and the choking phenomenon inside the ejector are too complicated to be completely understood. The conservation equations of mass, energy, and momentum, some gas dynamic equations, state equations, isentropic relations as well as some appropriate assumptions need to be used to assist in the description of the mixing process inside the ejector. Moreover, some factors that do not influence the flow significantly are neglected, so that the complexity of the governing equations can be reduced and the solution process is relatively simple and time-saving. The basic assumptions made before establishing mathematical models are as follows [35]:

1. The internal fluid of the ejector is stable.
2. The inner wall of the ejector is adiabatic.
3. The change in the fluid in the ejector is an isentropic process.

Based on the above assumptions, the following expressions are used [31]:

1. Laws of conservation of mass:

(10) $$\begin{array}{c} \frac{\partial \rho }{\partial t}+\frac{\partial \left({\rho u}_{i}\right)}{\partial x_{i}}=0 \end{array}$$

2. Law of conservation of energy:

(11) $$\frac{\partial \left(\rho E\right)}{\partial t}+\frac{\partial {(u}_{i}\left(\rho E+p)\right)}{\partial x_{i}}=\nabla \left(\alpha_{eff}\frac{\partial T}{\partial x_{i}}\right)+\nabla \left(u_{j}{(\tau }_{ij})\right)$$

3. Law of conservation of momentum:

(12) $$\begin{array}{c} \frac{\partial \left({\rho u}_{i}\right)}{\partial t}+\frac{\partial \left({\rho u}_{i}u_{j}\right)}{\partial x_{j}}=-\frac{\partial p}{\partial x_{i}}+\frac{\partial \tau_{ij}}{\partial x_{j}} \end{array}$$

(13) $$\begin{array}{c} \tau_{ij}{=\mu }_{eff}\left(\frac{\partial u_{i}}{\partial x_{j}}+\frac{\partial u_{j}}{\partial x_{i}}\right)-\frac{2}{3}\mu_{eff}\frac{\partial u_{k}}{\partial x_{k}}\delta_{ij} \end{array}$$

4. Droplet number density equation:

(14) $$\begin{array}{c} \frac{\partial \left(\rho n\right)}{\partial t}+\nabla \left(\rho un\right)=\rho I \end{array}$$

where ρ is the density, E is the total energy, u is the velocity, $\mu_{eff}$ is the dynamic viscosity coefficient, $\alpha_{eff}$ is the thermal conductivity, T is the static temperature.

### 3.4. CFD Settings

#### 3.4.1. Mesh

In CFD simulations, the quality of the mesh is critical for the solution process, and the axisymmetric two-dimensional model and three-dimensional model provide similar results for the ejector [19]. Considering computational efficiency, a two-dimensional axisymmetric mesh was used to simulate the ejector.

**Figure 3:**
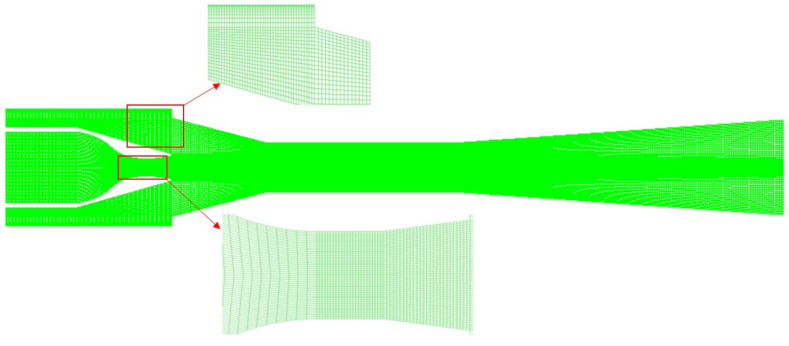
Mesh of the ejector.

In this study, an integrated computer engineering and manufacturing code (ICEM) is used to divide the ejector model into a quadrilateral mesh. ICEM is a professional computer-aided engineering (CAE) pre-processing software. ICEM can establish a high-quality structured mesh for complex models. The mesh is composed of quadrilateral cells, the details of the mesh are illustrated in Figure 3. It is noted that the mesh of the near-wall layer is refined and densified to capture the rapid gradient changes near the wall. The quality of the mesh evaluation function of the ICEM was used for the analysis. The proportion of the mesh orthogonal quality above 0.95 was 99.92%, and the number of angles between 85.5° and 90° was 87.62%. The mesh quality was excellent for the CFD simulations.

#### 3.4.2. Boundary Conditions and Turbulence Model Selection

In the simulation analysis, the primary and secondary inlets of the ejector were set as pressure inlets, and the outlet of the ejector was set as the pressure outlet. Smooth fixed wall surface with no-slip insulation. The boundary conditions are configured according to the operating conditions listed in Table 2. Relevant studies have shown that for the calculation and analysis of the wet steam operation process in the ejector, the turbulence model SST k-ω has better overall and local prediction ability than the other six turbulence models [36]. Therefore, this experiment used the turbulence model to simulate and analyze the ejector.

#### 3.4.3. Mesh Independence Verification

**Figure 4:**
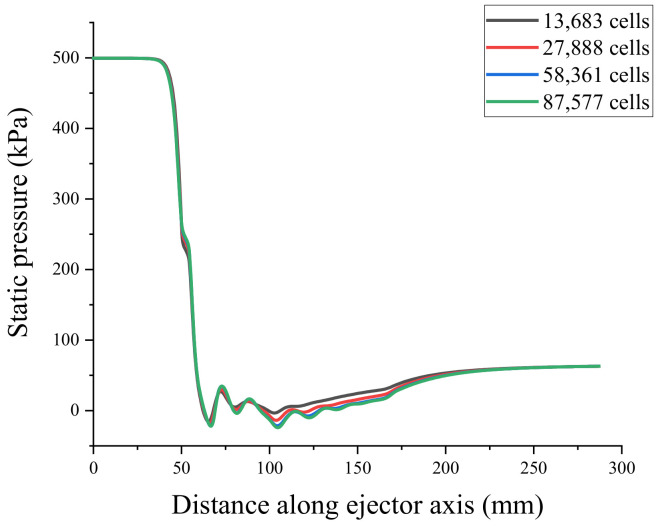
Axial static pressure curve.

**Figure 5:**
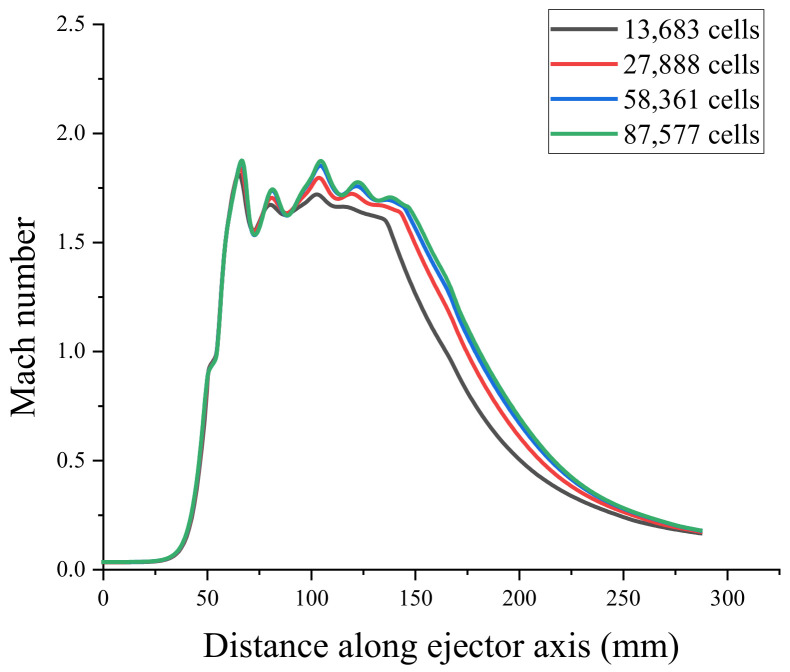
Axial Mach number curve.

**Figure 6:**
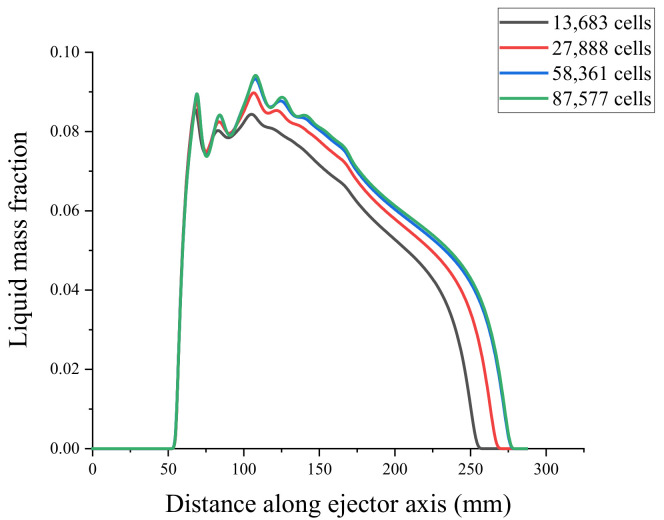
Axial liquid mass fraction.

**Figure 7:**
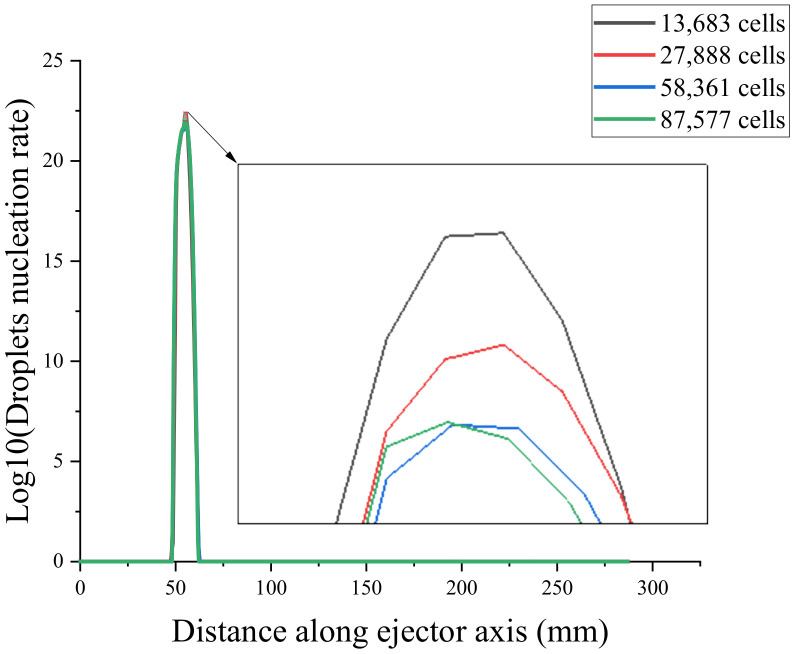
Axial droplet nucleation rate.

To verify the mesh independence and ensure that the number of meshes did not affect the simulation results of the ejector, the ejector model was divided into coarse mesh (13683), medium mesh (27888), fine mesh (58361), and very fine mesh (87577). Mesh independence verification using the axial Mach number and static pressure [31] is shown in Figure 4 and Figure 5. Because the non-equilibrium condensation process of the ejector was explored in this study, the axial liquid mass fraction and droplet nucleation rate were used to assist the verification of mesh independence, as shown in Figure 6 and Figure 7. According to these figures, the static pressure, Mach number, liquid mass fraction, and droplet nucleation rate curves of the fine and very fine meshes were consistent.

A small number of meshes will increase the area of a single mesh, which will weaken the accuracy of the CFD simulation analysis of the internal flow field distribution of the ejector. However, using an excessive number of meshes will require large amounts of computational resources, and there are problems such as slow convergence speed and many convergence steps in the solution process. In this study, a fine mesh (58361) was employed for CFD simulations based on mesh independence verification, considering the accuracy and efficiency of computing.

### 3.5. Experimental Verification

**Figure 8:**
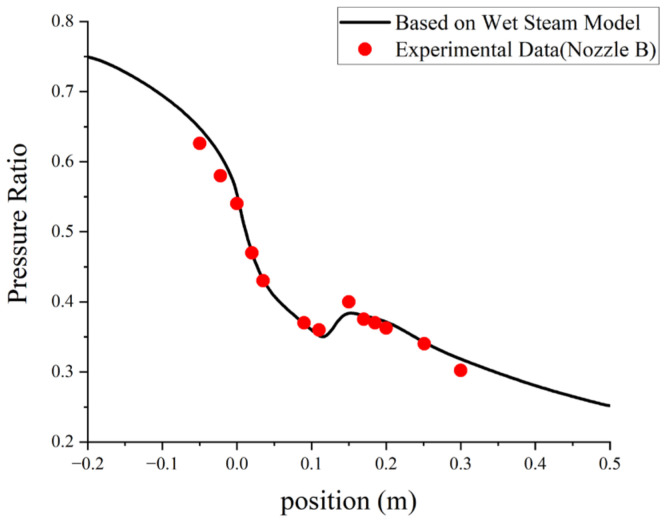
Numerical and experiment results of the static pressure along the axis of the Moore-B nozzle.

The primary nozzle in Moore-B [37] was selected to validate the wet steam model, the detailed geometries of the nozzle are shown in Table 3. The pressure inlet and pressure outlet were set at the nozzle inlet and outlet. The total pressure and temperature at the nozzle inlet were 25 kPa and 356.7 K, respectively. The profiles of the static pressure along the axis of the nozzle are shown in Figure 8. The maximum average relative error is 5.2%, and the total average relative error is 2.5%. As shown in Figure 8, the simulated pressure ratios along the nozzle axis are in good agreement with the experimental data. Thus, the wet steam model can be used to simulate the non-equilibrium condensation process inside the ejector.

**Figure 9:**
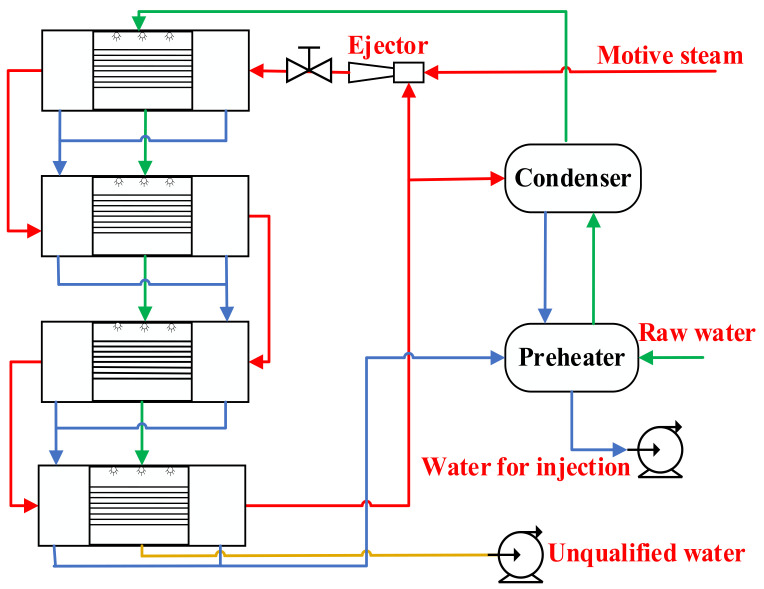
Distilled water preparation system for medical injection.

Multi-effect distillation technology was used in the distilled water preparation system for medical injection. Figure 9 shows a simplified process diagram. The process consisted of four horizontal tube falling film evaporators in series. The pressure gradient and gravity gradient between the evaporators are used to drive the raw water flow and evaporate, and the ejector is used to remove the final effect of steam for recycling to optimize energy usage. The raw water enters the first-effect evaporator after being preheated by the preheater and condenser and then sprays on the first-effect evaporation cross-tube through the liquid distribution device. Raw water flowed downward in the form of a thin film. Part of the water film absorbs the latent heat released by the condensation of power steam in the tube and then evaporates. Steam was used as a heating source for the second-effect evaporator through a foam capture device. The condensate water in the tube is discharged into the product water, while the remaining water flows into the next effect as feed water. The second, third, and fourth effects repeat the spray, evaporation, and condensation processes, respectively. The fourth-effect of the condensate converges with the first three effects of the product water, and then exchanges heat with the feed water in the condenser.

**Figure 10:**
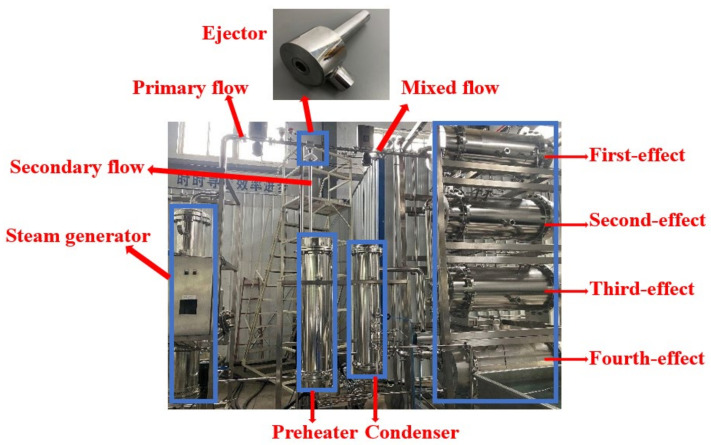
Distilled water preparation system for medical injection.

Figure 10 shows the equipment used for distilled water preparation. Table 4 lists the findings of ten groups of experimental tests. The relative errors are all within 10%, while the relative errors for the majority of the data are within 3%. The results of the CFD simulations are consistent with the results of the experimental tests. In the table, P_p_, P_s_, and P_b_ are the pressures of the primary inlet, secondary inlet, and outlet, respectively. T_p_, T_s_, and T_b_ are the temperatures of the primary inlet, secondary inlet, and outlet, respectively.

**Figure 11:**
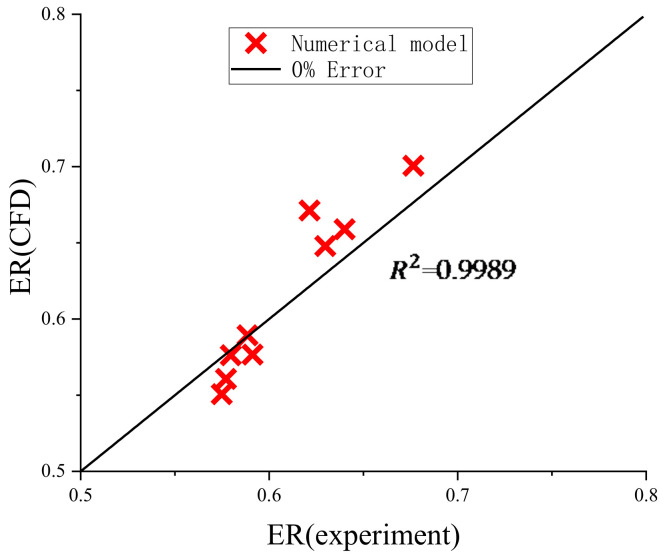
Comparison between CFD simulation and measurements.

The root means that the square method is used to evaluate the error between the CFD and experimental data:

(15) $$R^{2}=1-\frac{\sum_{i=1}^{n}{\left(a_{i}-p_{i}\right)}^{2}}{\sum_{i=1}^{n}{\left(p_{i}\right)}^{2}}$$

where $a_{i}$ is the experimental data point, and $p_{i}$ is the CFD data point. The simulation results shown in Figure 11 are slightly different from the experimental results, which may be owing to the simplified assumptions in the calculation process and the error of the experimental measurement.

## 4. Results and Discussion

### 4.1. Effect of Superheat on Ejector Performance

**Figure 12:**
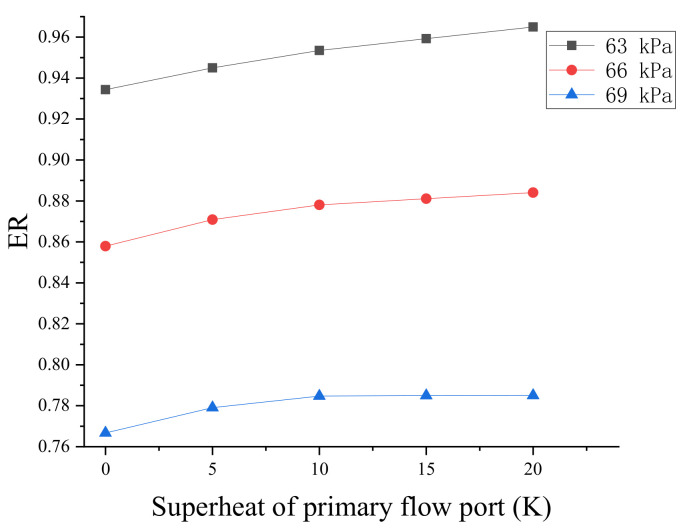
Effect of primary flow superheat on entrainment ratio.

Figure 12 depicts the curve of the entrainment ratio at different back pressures as the superheat level of the primary flow increases. As the superheat increases, the entrainment ratio gradually increases, and the entrainment ratio decreases as the backpressure increases. The effect of superheat on the entrainment rate is obvious when the superheat level is low, but it is less noticeable when the superheat level is high. The entrainment ratio increases by 3% for the superheat level of the primary flow increases from 0 to 20 K while the backpressure remains constant. The entrainment ratio decreases by 0.09 for every 3 kPa increase in the backpressure from 63 to 69 kPa.

**Figure 13:**
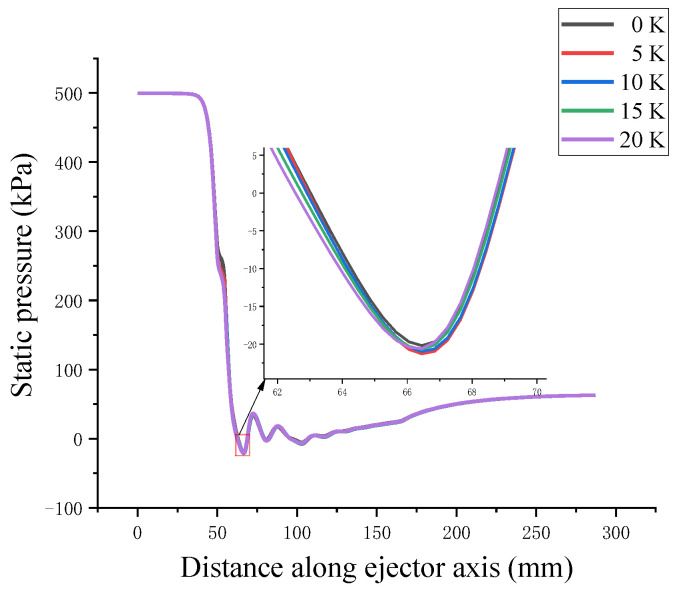
Effect of primary flow superheat on static pressure.

**Figure 14:**
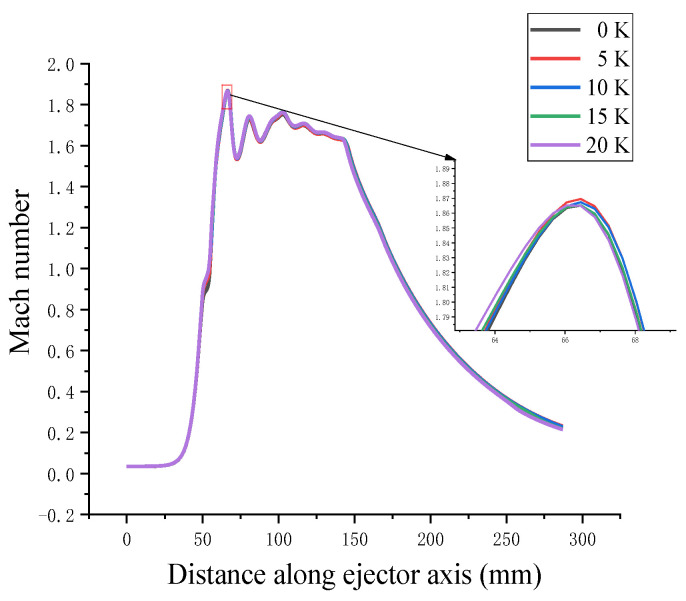
Effect of primary flow superheat on Mach number.

The axial static pressure curve in Figure 13 shows the pressure change process of the high-pressure steam jetting low-pressure secondary flow on the axis of the supersonic ejector. The primary flow achieves supersonic velocity at the nozzle exit through the Laval nozzle, and there is violent energy and momentum exchange in the mixing chamber with the secondary flow. Turbulence occurs when there is a rapid change in the velocity. A shock wave was generated in the constant-area chamber. The fluid velocity decreased rapidly, whereas the fluid pressure increased. The kinetic energy was then converted into pressure potential energy through the diffusion chamber, completing the pressurization and deceleration processes. The figure shows that the latent heat released by droplet nucleation affected the condensation shock wave. The Mach number in the fluid is affected by the superheat level of the primary flow, as shown in Figure 14. The pressure potential energy is converted to kinetic energy by the law of conservation of energy, and the change in Mach number is opposed to the trend of static pressure.

**Figure 15:**
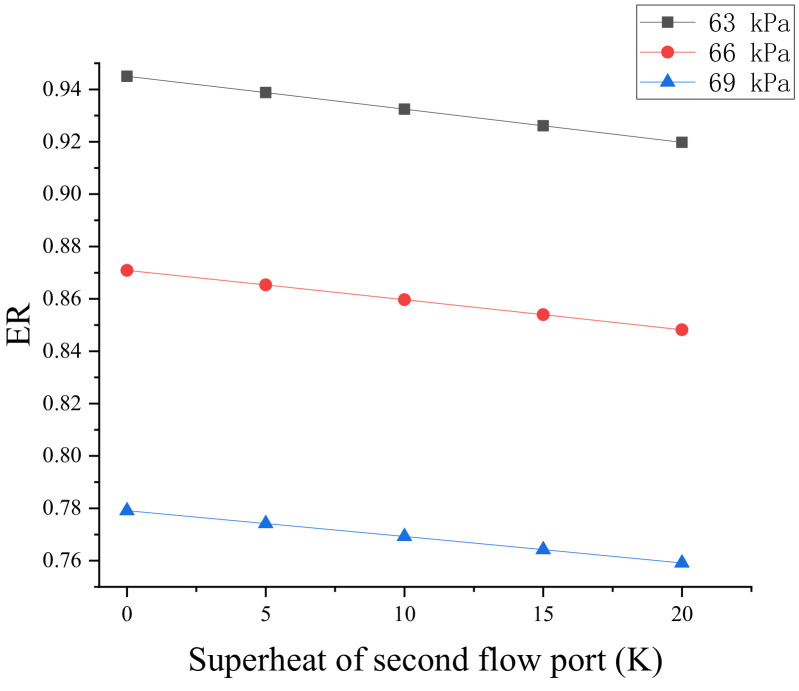
Effect of secondary flow superheat on entrainment ratio.

The influence of secondary flow superheat on the entrainment ratio is shown in Figure 15. Under varied backpressure circumstances (63, 66, and 69 kPa), the entrainment ratio decreased linearly as the secondary flow superheat increased. The entrainment ratio decreased by 0.5% for every 5 K increase in the superheat level. The temperature difference between the primary and secondary flows is smaller, and the pressure differential between the secondary flow and primary flow nozzle exit is also smaller, resulting in a lower entrainment ratio in the ejector. As a result, in engineering applications, the secondary flow superheat should be minimized as much as possible to ensure the operational efficiency of the ejector.

**Figure 16:**
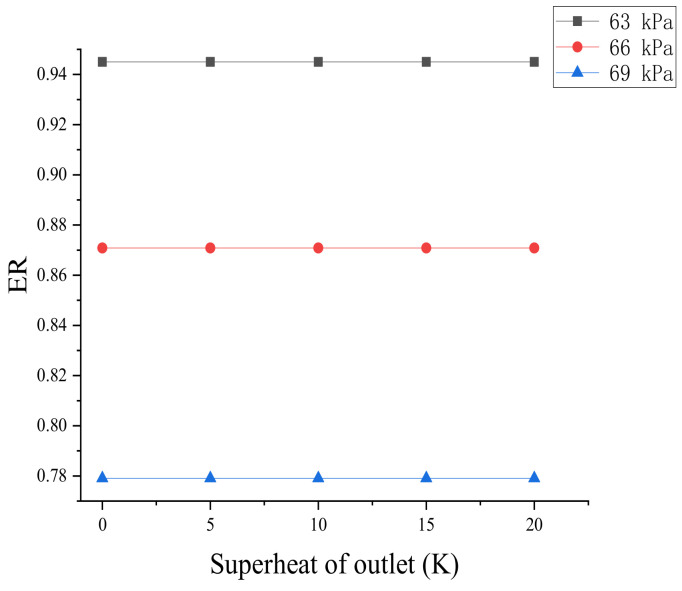
Effect of outlet superheat on entrainment ratio.

Figure 16 shows the effect of outlet flow superheat on the entrainment ratio. The entrainment ratio almost did not change under different backpressure conditions (63, 66, and 69 kPa) when the ejector outlet superheat raised from 0 to 20 K.

### 4.2. Effect of Superheat on Non-Equilibrium Condensation

**Figure 17:**
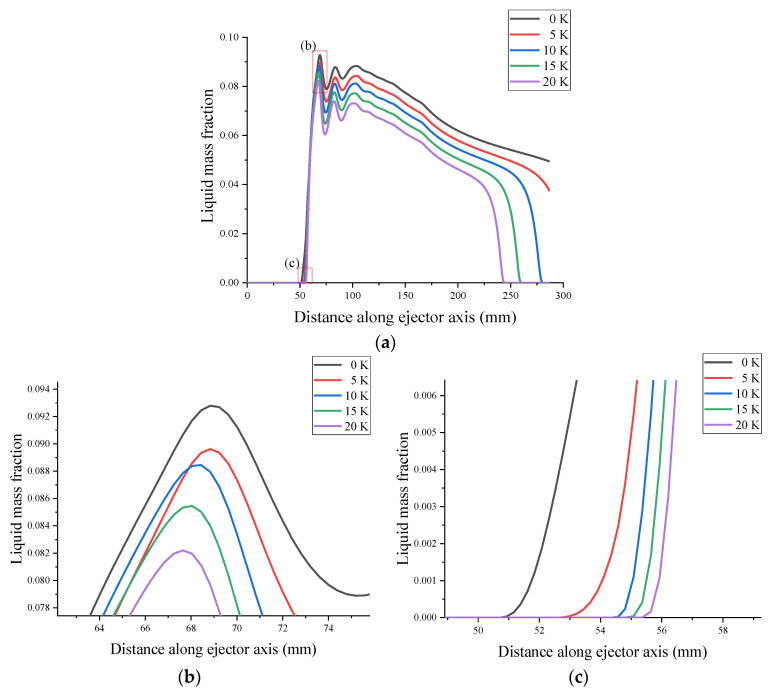
Effect of primary flow superheat on liquid mass fraction. (**a**) shows liquid mass fraction of ejector, (**b**) shows the peak liquid mass fraction of ejector, (**c**) shows the starting position of ejector.

Primary superheated wet steam at 0, 5, 10, 15, and 20 K was employed for the simulation analysis, while the secondary inlet and outlet conditions of the ejector were constant. The axial liquid mass fraction obtained from the CFD simulation is shown in Figure 17. Figure 17a shows that compared with the 0 K superheat primary flow, the 20 K superheat causes the condensed droplets to disappear and is shifted forward to 42 mm from the ejector exit, whereas the liquid mass fraction decreases by 20% in the constant-pressure mixing chamber and constant-area mixing chamber. Figure 17b shows the distribution of the liquid mass fraction reaching its peak at various primary superheat levels, with the peak liquid mass fraction decreasing as the superheat level increases. Figure 17c shows that the superheat of the primary flow increases by 20 K, and the nucleation starting position of the liquid moves 5 mm downstream of the ejector.

**Figure 18:**
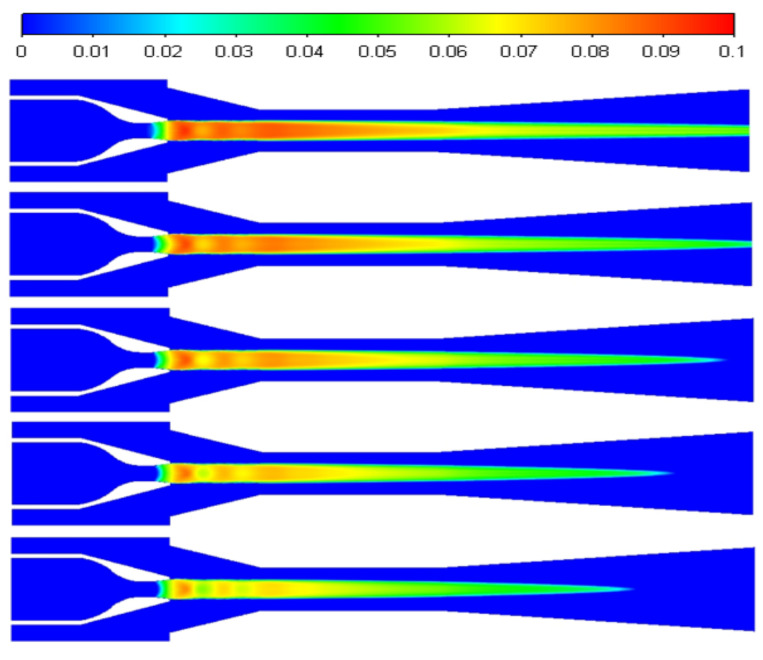
The contours of liquid mass fraction at different primary flow superheat levels.

The distribution of liquid mass fractions at 0, 5, 10, 15, and 20 K is shown in Figure 18 from top to bottom. The intensity of non-equilibrium condensation in the ejector is inhibited as the primary superheat increases, the liquid mass fraction in the ejector decreases, and the liquid mass fraction in the ejector’s diffusion chamber decreases significantly.

**Figure 19:**
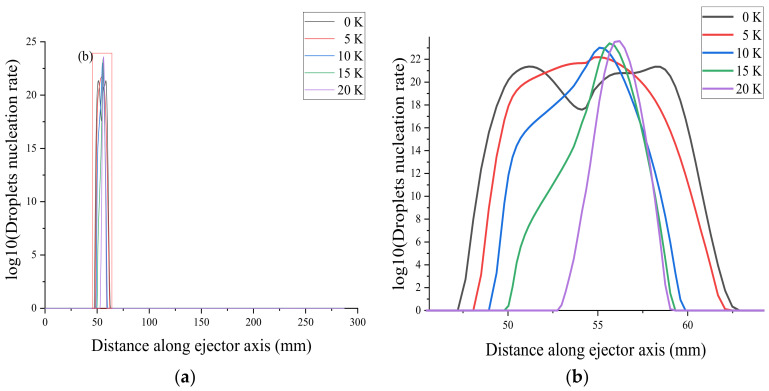
Effect of primary flow superheat on the droplet nucleation rate. (**a**) shows the droplets nuclearation rate of ejector, (**b**) shows the local droplets nuclearation rate of ejector.

The starting position of droplet nucleation in the ejector was delayed by 5 mm as the primary flow superheat increases from 0 to 20 K, as shown in Figure 19a. The maximum nucleation rate point is called the Wilson point [38]. As the superheat increases, the two Wilson points come closer together and finally merge when the superheat level reaches 5 K, as shown in Figure 19b. As the superheat increases, the starting position of the droplet nucleation rate shifts backward, and the ending position shifts forward.

**Figure 20:**
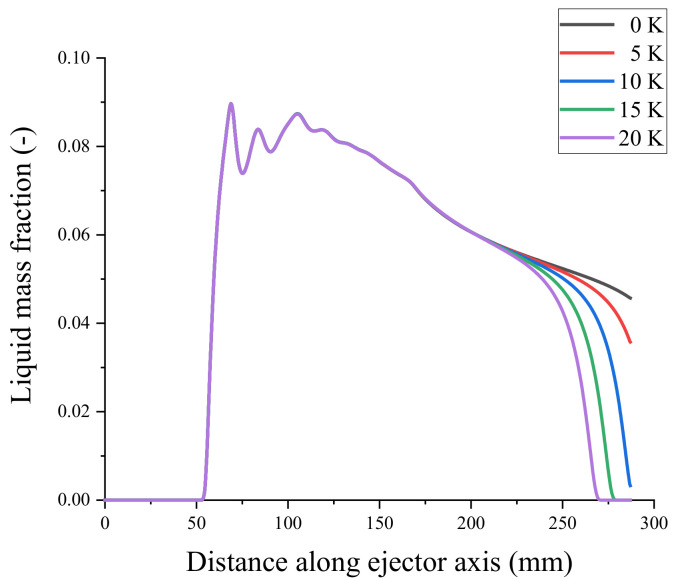
Effect of secondary flow superheat on liquid mass fraction.

**Figure 21:**
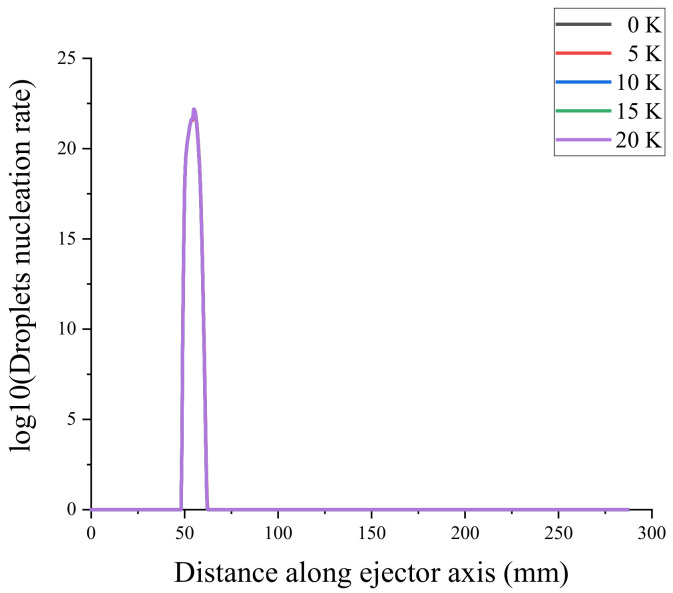
Effect of secondary flow superheat on the droplet nucleation rate.

**Figure 22:**
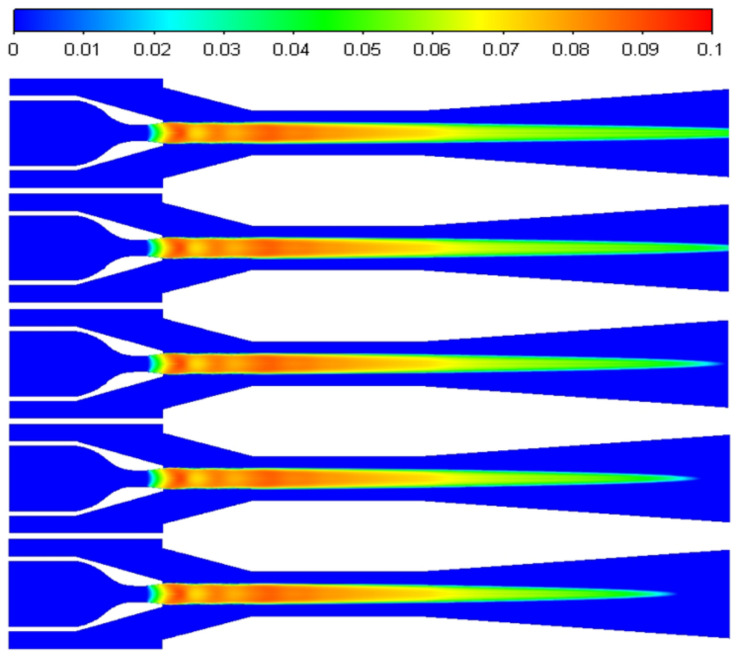
The contours of liquid mass fraction at different secondary flow superheat levels.

Under constant primary flow inlet and outlet conditions of the ejector, superheat levels of 0, 5, 10, 15, and 20 K were used for the secondary flow steam, resulting in the axial liquid mass fraction distribution shown in Figure 20. The effects of varying the secondary flow superheat levels on the droplet nucleation rate are shown in Figure 21. From top to bottom, Figure 22 shows the liquid mass fraction contours for the secondary flow superheat levels of 0, 5, 10, 15, and 20 K. According to these figures, the secondary flow superheat does not affect the liquid mass fraction distribution of the primary nozzle, mixing chamber, and constant-area mixing chamber but only affects the liquid mass fraction of the diffusion chamber. The rise in the secondary inlet superheat results in a decrease in the liquid mass fraction in the diffusion chamber of the ejector. The liquid in the ejector evaporates fully 16 mm from the outlet as the superheat increases from 0 to 20 K.

**Figure 23:**
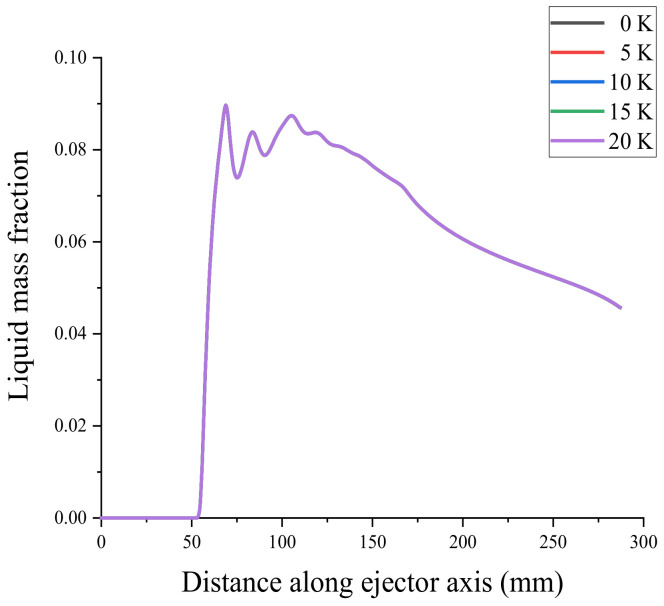
Effect of outlet superheat on liquid mass fraction.

**Figure 24:**
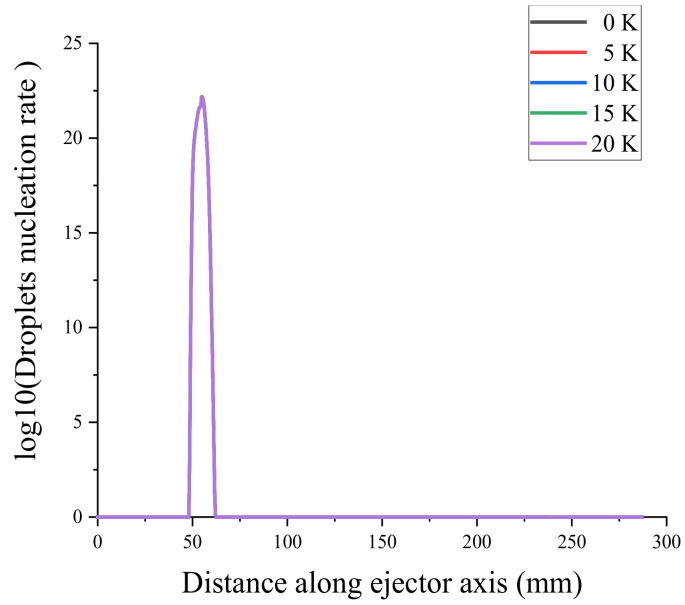
Effect of outlet superheat on the droplet nucleation rate.

The superheat levels at the exit of the ejector were set to 0, 5, 10, 15, and 20 K, respectively. The liquid mass fraction was obtained, as illustrated in Figure 23, and the droplet nucleation rate curve is shown in Figure 24. The output superheats of the ejector increase from 0 to 20 K, while the ejector’s liquid mass fraction and droplet nucleation rate remain essentially constant.

## 5. Conclusions

This study investigated the effect of a three-port superheat in a supersonic ejector used in a distilled water preparation system for medical injection on the ejector entrainment ratio and the non-equilibrium condensation phenomenon using the wet steam model. Key parameters such as the ejector entrainment ratio, droplet nucleation rate, and liquid mass fraction are investigated both theoretically and experimentally. The following conclusions were drawn:

The primary flow superheat increases the ejector entrainment ratio while weakening the nonequilibrium condensation phenomenon. The primary flow superheat of 20 K increases the entrainment ratio by 3%. The non-equilibrium condensation starting location moves backward by 5 mm, and the liquid mass fraction decreases by 20% in the constant-pressure and constant-area mixing chambers.

The superheat of the secondary flow reduces the ejector entrainment ratio and affects condensation. For every 5 K increase in the secondary flow superheat, the entrainment ratio decreases by 0.5%, and the liquid mass fraction in the diffusion chamber decreases.

The effect of the exit superheat on the entrainment ratio and non-equilibrium condensation is negligible.

## Figures and Tables

**Table 1:** Structural parameters of ejector.

Item	Value	Unit
Nozzle throat length	5.00	mm
Nozzle throat diameter	6.50	mm
Nozzle inlet diameter	26.06	mm
Nozzle outlet diameter	8.22	mm
Suction chamber length	61.28	mm
Suction chamber diameter	43.38	mm
Constant-pressure inlet diameter	36.21	mm
Constant-pressure length	35.36	mm
Constant-area length	68.36	mm
Constant-area diameter	17.84	mm
Diffusion chamber diameter	34.93	mm
Diffusion chamber length	122.00	mm

**Table 2:** Operating conditions of ejector.

Item	Temperature (K)	Pressure (kPa)
Primary inlet	432.05	500.00
Secondary inlet	376.35	12.40
Outlet	388.50	63.00

**Table 3:** Key geometric parameters of Moore-B nozzle.

X (mm)	−250	−200	0	500
Y (mm)	±56.35	±56.35	±50	±72

**Table 4:** Comparison between CFD simulation and measured data.

Times	T_p_(K)	P_p_(kPa)	T_s_(K)	P_s_(kPa)	T_b_(K)	P_b_(kPa)	ER_experiment_	ER_CFD_	Error (%)
1	431.95	509.0	376.35	12.4	388.75	71.1	0.6297	0.6478	2.87
2	435.15	557.2	375.85	11.1	388.85	73.7	0.6214	0.6713	8.03
3	434.65	542.8	374.65	6.9	387.35	65.6	0.5748	0.5507	4.19
4	436.35	575.6	375.25	7.8	389.35	75.1	0.5796	0.5762	−0.59
5	436.35	575.6	375.05	7.0	389.15	74.1	0.5885	0.5893	0.14
6	435.25	552.0	373.55	2.6	386.45	60.9	0.6765	0.7006	−3.56
7	435.75	565.8	375.85	10.4	389.65	77.3	0.5771	0.5607	−2.84
8	436.45	577.8	374.04	3.5	388.45	70.3	0.5911	0.5767	−2.44
9	436.15	570.9	376.85	14.6	389.85	78.8	0.6400	0.6589	2.95
10	433.85	530.9	373.15	0.5	385.65	70.9	0.3217	0.3074	4.44

## Data Availability

Data sharing not applicable.
